# Supplementary material for: Analysis of a Real-World Cohort of Metastatic Breast Cancer Patients Shows Circulating Tumor Cell Clusters (CTC-clusters) as Predictors of Patient Outcomes
Source: Cancers (Basel). 2020 Apr 29;12(5):1111. doi: 10.3390/cancers12051111 (PMC7281711; doi:10.3390/cancers12051111)
Supplement: Supplementary file 1 [file cancers-12-01111-s001.pdf]

Analysis of A Real-world Cohort of Metastatic Breast Cancer Patients Shows Circulating Tumor Cell Clusters (CTC-clusters) as A Predictor of Patient Outcomes

Clotilde Costa , Laura Muinelo-Romay, Victor Cebey-López, Thais Pereira-Veiga, Inés Martínez-Pena, Manuel Abreu, Alicia Abalo, Ramón M. Lago-Lestón, Carmen Abuín, Patricia Palacios, Juan Cueva, Roberto Piñeiro and Rafael López-López

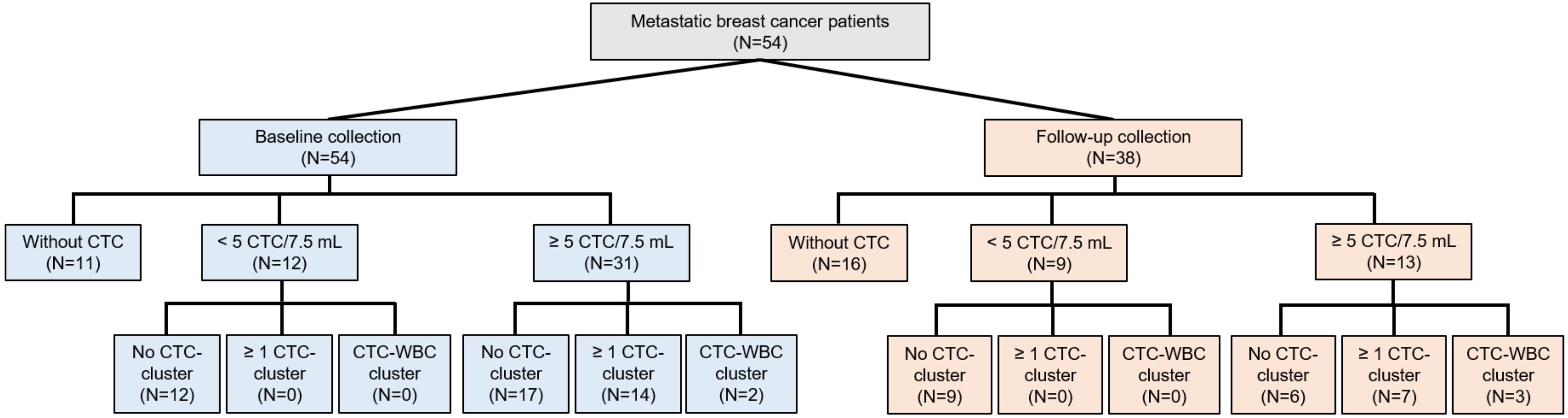

Figure S1. Study design. CONSORT flow diagram of study cohort and patient distribution at times of blood draw according to the presence or not of CTCs, CTC-clusters, and CTC-WBC clusters (N indicates the number of patients).

cancers MDPI

a

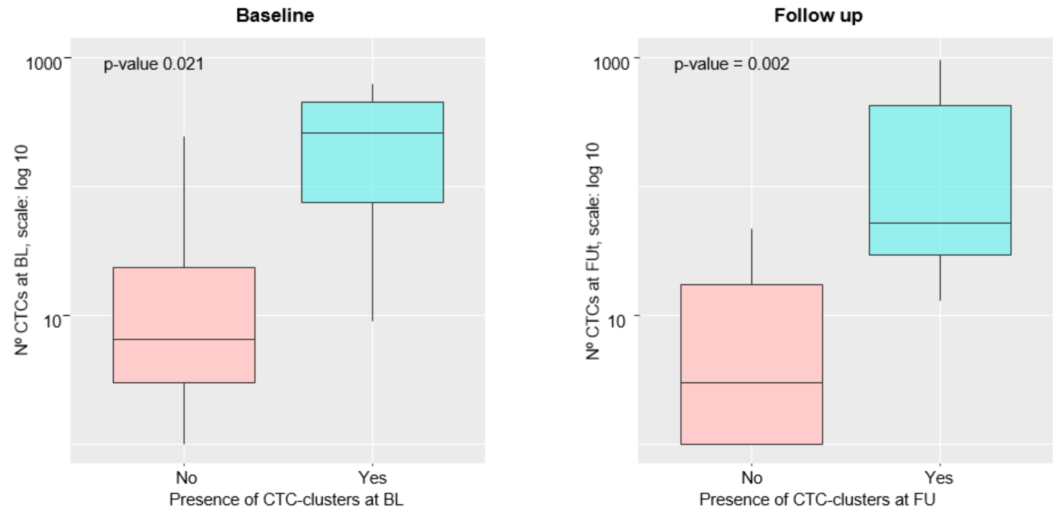

b

Baseline	CTC-clusters=No	CTC-clusters=Yes
< 5 CTC	23	0
≥ 5 CTC	17	14

Follow up	CTC-clusters=No	CTC-clusters=Yes
< 5 CTC	25	0
≥ 5 CTC	6	7

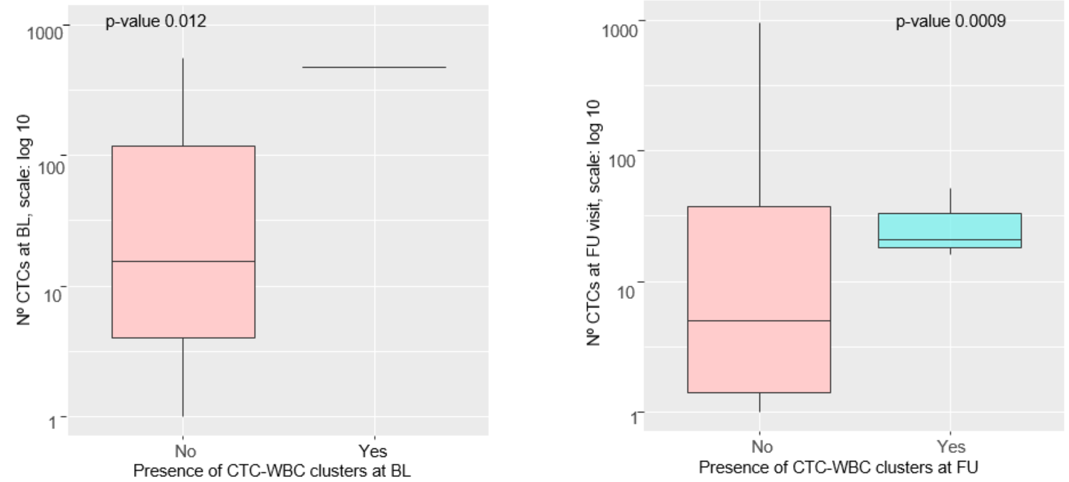

Baseline	CTC-WBC clusters=No	CTC-WBC clusters=Yes
< 5 CTC	23	0
≥ 5 CTC	29	2

Follow up	CTC-WBC clusters=No	CTC-WBC clusters=Yes
< 5 CTC	25	0
≥ 5 CTC	10	3

Figure S2. Association between CTC count and the presence of CTC-clusters or CTC-WBC clusters at baseline and follow up. Box plots showing the association between the number of CTCs and the presence or absence of CTC-clusters at baseline (BL) and follow up (FU) (a). Box plots showing the association between the number of CTCs and the presence or absence of CTC-WBC clusters at baseline (BL) and follow up (FU) (b). Associations were assessed using Pearson's chi-squared test.

Table S1. Study cohort analysis of CTCs and CTC-clusters.

Baseline	n= 54	Percent (%)	Mean	Min-Max
CTCs	43	79.2	183.1	1-1970
CTCs ≥ 5	31	57.4	253.1	5-1970
CTC-cluster ≥ 1	14	25.9	4.28	1-14
CTC-WBC-cluster ≥ 1	2	3.7	7.5	2-13
**Follow up**	n= 38			
CTCs	22	57.9	95.6	1-969
CTCs ≥ 5	13	34.2	160.6	5-969
CTC-cluster ≥ 1	7	18.4	12.71	1-74
CTC-WBC-cluster ≥ 1	3	7.9	2.0	2-2

Table S2. Distribution of CTCs and CTC-clusters in different BC subtypes.

**Variable**	**Total**	**BC subtypes**				***p* Value**
		**HR+HER2- (*n*= 27)**	**HER2+ (*n*= 11)**	**HR-HER2- (*n*= 15)**	**Undet. (*n*= 1)**	
Baseline CTCs						
< 5 CTCs/7.5 ml	23 (100.0)	12 (52.2)	4 (17.4)	7 (30.4)	0 (0)	0.861
≥ 5 CTCs/7.5 ml	31 (100.0)	15 (48.4)	7 (22.6)	8 (25.8)	1 (3.2)	
Baseline CTC-clusters						
No	40 (100.0)	21 (52.5)	7 (17.5)	11 (27.5)	1 (2.5)	0.668
Yes	14 (100.0)	6 (42.9)	4 (28.6)	4 (28.6)	0 (0)	
2-3 cells	12 (100.0)	6 (50)	3 (25)	3 (25)	0 (0)	
≥ 4 cells	2 (100.0)	0 (0)	1 (50)	1 (50)	0 (0)	
Baseline CTC-WBC clusters						
No	52 (100.0)	26 (50)	10 (19.2)	15 (28.8)	1 (1.9)	0.488
Yes	2 (100.0)	1 (50)	1 (50)			
		**(*n*= 19)**	**(*n*= 9)**	**(*n*= 9)**	**(*n*= 1)**	
Follow up CTCs						
< 5 CTCs/7.5 ml	25 (100.0)	11 (44)	7 (18.4)	6 (24)	1 (4)	0.583
≥ 5 CTCs/7.5 ml	13 (100.0)	8 (61.5)	2 (15.4)	3 (23.1)	0 (0)	
Follow up CTC-clusters						
No	31 (100.0)	15 (48.4)	9 (29.0)	6 (19.4)	1 (3.2)	0.184
Yes	7 (100.0)	4 (57.1)	0 (0)	3 (42.9)	0 (0)	
2-3 cells	5 (100.0)	3 (60)	0 (0)	2 (40)	0 (0)	
≥ 4 cells	2 (100.0)	1 (50)	0 (0)	1 (50)	0 (0)	
Baseline CTC-WBC clusters						
No	35 (100.0)	17 (48.6)	8 (22.9)	9 (25.7)	1 (2.9)	0.590
Yes	3 (100.0)	2 (66.7)	1 (33.3)	0 (0)	0 (0)	

Abbreviations: CTCs circulating tumor cells, HR hormone receptors, HER2 human epidermal growth factor receptor 2, Undet. undetermined.

Table S3. Multivariable Cox regression analysis of prognostic variables included in the clinicopathological model.

Variable	PFS		OS	
	**HR (95% CI)**	***p* Value**	**HR (95% CI)**	***p* Value***
Age at baseline				
< 65	1.0		1.0	
≥ 65	0.54 (0.24-1.2)	0.15	0.59 (0.23-1.5)	0.27
ECOG				
Ordinal scale (0. 1. 2)	0.73 (0.4-1.3)	0.36	0.82 (0.4-1.6)	0.56
Number of metastatic sites				
< 3	1.0		1.0	
≥ 3	1.7 (0.8-3.5)	0.18	2.0 (0.58-4.7)	0.12
Site of metastasis				
Non-visceral	1.0		1.0	
Visceral	2.5 (0.94-6.6)	0.067	5.4 (1.2-23)	0.026
Subtype				
HR+HER2-	1			
HER2+	1.38 (0.48-3.9)	0.547	0.85 (0.25-3.5)	0.94
HR-HER2-	1.63 (0.7-3.8)	0.25	1.9 (0.7-4.7)	0.16
Treatments				
Chemotherapy	1		1	
Hormone	0.13 (0.03-0.57)	0.006	0.10 (0.01-0.79)	0.028
Targeted	1.41 (0.48-4.16)	0.53	1.14 (0.3-3.9)	0.829

Abbreviations: HR hazard ratio and hormone receptors, PFS progression-free survival, OS overall survival, CI confidence interval, ECOG Eastern Cooperative Oncology Group, HER2 human epidermal growth factor receptor 2.

* Adjusted for age, ECOG, subtype, number of metastatic sites, site of metastasis and treatments.

Table S4. Cox regression analysis for CTC count ≥ 20 versus < 20, and presence versus absence of CTC-clusters.

Variables	Total	Events, *n* (%)	HR (95% CI)	*p* Value	HR (95% CI)*	*p* Value*
	Baseline					
Associated with OS						
≥ 5 vs < 5 CTCs	31	17 (54.8)	3.15 (1.16-8.55)	0.024	3.33 (1.14-9.73)	0.027
≥ 20 vs < 20 CTCs	21	13 (57.1)	3.53 (1.50-8.31)	0.0037	11.92 (2.79-50.93)	0.0008
Joint effect of baseline CTC and CTC-clusters						
Associated with OS						
< 20 CTCs	33	10 (30.3)	1.0		1.0	
≥ 20 CTCs without CTC-cluster	8	3 (37.5)	1.61 (0.43-6.04)	0.478	11.9 (1.5-94.7)	0.019
≥ 20 CTCs, ≥ 1 CTC-cluster	13	10 (76.9)	5.5 (2.19-13.76)	0.0002	11.9 (2.69-52.6)	0.0010

Abbreviations: CTC circulating tumor cell, OS overall survival, HR hazard ratio, CI confidence interval.
* Adjusted for age, ECOG, subtype, number of metastatic sites, site of metastasis and treatments.

Table S5. **Longitudinal changes in CTCs and CTC-clusters on relation to PFS and OS.**

**Variable**	**Total**	**Events, *n* (%)**	**HR (95% CI)**	***p* Value**	**HR (95% CI)[#]**	***p* Value[#]**
Associated with PFS						
No CTC	28	7 (25.0)	1.00		1.00	
1-4 CTC without CTC-cluster	21	10 (47.6)	1.39 (0.52-3.72)	0.50	1.53 (0.56-4.61)	0.45
≥ 5 CTC without CTC-cluster	25	14 (56.0)	1.82 (0.71-4.61)	0.21	2.00 (0.73-5.43)	0.17
≥ 5 CTC ≥ 1 CTC-cluster	22	16 (72.7)	3.99 (1.64-9.73)	0.0022	4.65 (1.79-12.61)	0.0024
1-4 CTC without CTC-cluster			1.00		1.00	
≥ 5 CTC without CTC-cluster			1.31 (0.57-3.01)	0.52	1.227 (0.40-3.41)	0.69
≥ 5 CTC ≥ 1 CTC-cluster			2.88 (1.26-6.55)	0.0115	2.863 (1.00-8.15)	0.0489
Associated with OS						
No CTC	28	3 (10.7)	1.00		1.00	
1-4 CTC without CTC-cluster	21	6 (28.7)	1.62 (0.38-6.79)	0.51	3.32 (0.62-17.62)	0.16
≥ 5 CTC without CTC-cluster	25	12 (48.0)	4.01 (1.12-14.34)	0.0324	7.38 (1.68-32.43)	0.0081
≥ 5 CTC ≥ 1 CTC-cluster	22	15 (68.1)	7.91 (2.28-27.37)	0.0010	13.93 (3.22-60.25)	0.0004
1-4 CTC without CTC-cluster			1.00		1.00	
≥ 5 CTC without CTC-cluster			2.45 (0.86-6.99)	0.093	2.22 (0.64-7.68)	0.2051
≥ 5 CTC ≥ 1 CTC-cluster			4.84 (1.75-13.41)	0.0023	4.42 (1.25-15.63)	0.0209

Abbreviations: CTC circulating tumor cell, PFS progression-free survival, OS, overall survival, HR hazard ratio, CI confidence interval.

[#] Adjusted for age, ECOG, subtype, number of metastatic sites, site of metastasis and treatments.
